# Hospital Length of Stay and Associated Factors in Patients with Oral Cavity Cancer in Germany: A Retrospective Multicenter Analysis of Inpatient Administrative Data

**DOI:** 10.3390/reports9030296

**Published:** 2026-09-02

**Authors:** Lisa Lotta Cirkel, Isabel Klein, Karel Kostev

**Affiliations:** 1 Department of Cariology, Periodontology, and Endodontology, Dental Clinic, Justus Liebig University, 35392 Giessen, Germany; 2 Real World Data Analytics, IQVIA, 60597 Frankfurt, Germany; 3 Marburg University, University Hospital, 35043 Marburg, Germany; 4 Epidemiology, IQVIA, 60549 Frankfurt, Germany

**Keywords:** oral cavity cancer, length of stay, hospitalization, Germany, comorbidity, inpatient care, health services research

## Abstract

Background: Oral cavity cancer is a clinically relevant subgroup of head and neck malignancies and is associated with substantial treatment burden and healthcare utilization. Hospital length of stay (LOS) is an important indicator of inpatient resource use and complexity of care, yet large multicenter data from Germany are limited. Methods: This retrospective multicenter analysis used anonymized inpatient administrative data from 49 German hospitals; eligible oral cavity cancer hospitalizations were contributed by 34 of these hospitals. Adult inpatient hospitalizations (≥18 years) with malignant neoplasms of the oral cavity, defined using ICD-10-GM codes C00–C06, recorded between January 1 2019 and 31 December 2024 were included. The primary outcome was hospital LOS in days. Multimorbidity was quantified using the van Walraven-weighted Elixhauser Comorbidity Score. Prolonged hospitalization was defined as LOS ≥ 7 days and LOS ≥ 14 days. Associations between demographic, clinical, and treatment-related variables and LOS were examined using multivariable Poisson regression models. Because overdispersion was present, a negative binomial mixed model was additionally fitted as a sensitivity analysis. To account for inter-hospital variability, hospital was included as a random intercept in all multivariable models. Associations with prolonged LOS were analyzed using multivariable logistic regression models. All analyses were performed at the hospitalization level. Results: A total of 3957 inpatient hospitalizations for oral cavity cancer were included. Mean age was 65.6 years, and 66.2% of hospitalizations involved male patients. The median LOS was 6 days (interquartile range [IQR] 3–13; mean 10.2 days, standard deviation 11.8). Overall, 49.4% of hospitalizations had an LOS ≥ 7 days and 23.5% had an LOS ≥ 14 days. Older age, particularly >80 years, and higher comorbidity burden were associated with longer LOS (adjusted Poisson rate ratio [RR] for age > 80 years 1.17, 95% CI 1.13–1.21; high comorbidity burden RR 1.58, 95% CI 1.54–1.63). Several treatment-related variables, including surgical procedures in the oral and facial region, lymphatic system operations, blood transfusions, and complex intensive care treatment, were associated with prolonged hospitalization (e.g., blood transfusion RR 1.80, 95% CI 1.76–1.85; complex intensive care RR 1.70, 95% CI 1.65–1.75). Chemotherapy-related hospitalizations were associated with shorter LOS. Conclusions: LOS varied substantially across inpatient hospitalizations for oral cavity cancer in Germany. Older age, higher comorbidity burden, and markers of more complex inpatient treatment were associated with extended hospital stay. These findings may help identify hospitalizations at increased risk of prolonged LOS and inform inpatient planning and resource allocation.

## 1. Introduction

Oral cavity cancer (OCC) is an important subgroup of head and neck malignancies and contributes substantially to the global cancer burden [1,2]. In 2020, approximately 377,000 new cases of oral squamous cell carcinoma (OSCC) were diagnosed worldwide, and OCC continues to represent a clinically relevant malignancy in Germany [3,4]. The prognosis depends heavily on the UICC stage at diagnosis, with the highest relative 5-year survival rate observed in stage I and survival rates being significantly lower in advanced disease. In the cancer report published by the Robert Koch Institute (RKI), the relative 5-year survival rate for Stage I is reported as 85% for women and 70% for men [4]. Owing to the frequently asymptomatic course of early disease, most patients present at advanced stages, resulting in persistently limited survival gains and a substantial unmet medical need.

Management of OCC is therefore predominantly driven by surgery-based multimodal treatment strategies, frequently involving extensive tumor resections, neck dissection, and complex reconstructive procedures [5,6,7,8,9]. At the same time, comorbidity burden is increasingly recognized as an important factor associated with short-term surgical outcomes and hospitalization burden in older patients with head and neck cancer [10,11]. Administrative data also allow multimorbidity to be captured using established summary measures such as the van Walraven weighted Elixhauser Comorbidity Score [12]. In this context, hospitalization outcomes reflect not only surgical complexity but also patient vulnerability, comorbidity burden, and postoperative recovery trajectories, rendering perioperative metrics such as hospital length of stay (LOS) clinically meaningful indicators of treatment intensity and overall burden of care [5,6,7,8,9,11,13,14].

Despite the expanding literature, most previous LOS studies have focused on selected surgical cohorts or on healthcare systems outside Germany [5,6,8,9]. In Germany, administrative inpatient data have already been used to study LOS in several other disease areas, including depression, intracranial injuries, cardiovascular diseases, and orthopedic conditions [15,16], but comparable multicenter analyses for OCC are scarce. In particular, the influence of age, comorbidity burden, tumor site, and treatment-related variables on LOS in real-world inpatient hospitalizations for OCC has not yet been sufficiently described in a multicenter German setting.

Understanding factors associated with prolonged LOS in OCC is clinically relevant because extended hospitalization is associated with increased healthcare costs, postoperative morbidity, resource utilization, delayed rehabilitation, and reduced hospital capacity. Identifying patient- and treatment-related determinants of prolonged LOS may therefore support inpatient resource planning, discharge coordination, and optimization of inpatient care pathways.

The aim of this multicenter cross-sectional study was therefore to describe hospital LOS among adult inpatient hospitalizations for OCC in Germany and to identify demographic, clinical, and treatment-related factors associated with longer inpatient stay.

## 2. Methods

### 2.1. Data Source

This retrospective multicenter analysis used anonymized inpatient administrative data provided by IQVIA from 49 hospitals in Germany. The dataset includes approximately 2,143,071 hospitalizations recorded between 1 January 2019 and 31 December 2024. The full source database of 49 hospitals encompasses a diverse spectrum of hospital types, including university medical centers, maximum-care and standard-care hospitals, primary care institutions, and specialized facilities; the care level and hospital type of the individual centers that contributed OCC hospitalizations were not available in the anonymized dataset. Participating hospitals represented different hospital care levels and geographic regions across Germany. All hospitals included in the database were screened for eligible OCC hospitalizations during the study period. Eligible OCC hospitalizations were contributed by 34 of the 49 hospitals; 13 of these contributed more than 10 OCC hospitalizations each. This distribution likely reflects differences in institutional specialization, case volume, and treatment focus across participating centers.

All contributing hospitals regularly submit their inpatient case data to the Institute for the Hospital Remuneration System (InEK) in compliance with §21 of the German Hospital Compensation Act (KHEntgG). Data transmission follows a standardized reporting structure. Prior to submission, all records are comprehensively anonymized in accordance with data protection regulations, with patient- and case-specific identifiers removed or substituted. The database contains anonymized individual hospitalization-level records, with each inpatient episode represented as a separate observation. Therefore, the unit of analysis was the hospitalization rather than the unique patient. Statutory case reporting to the Institute for the Hospital Remuneration System (InEK) under §21 KHEntgG is distinct from the de-identified IQVIA research data product analyzed here, in which direct and indirect patient identifiers remained unavailable; consequently, repeat admissions by the same individual could not be identified, and potential within-patient correlation could not be modeled.

### 2.2. Study Population

The study population comprised inpatient cases of adults aged ≥18 years with a diagnosis of malignant neoplasms of the oral cavity, including lip (ICD-10: C00), tongue (ICD-10: C01–C02), gum (ICD-10: C03), floor of mouth (ICD-10: C04), palate (ICD-10: C05), and other or unspecified parts of the mouth (ICD-10: C06), recorded between January 2019 and December 2024. Lip cancer (ICD-10: C00) was included because ICD-10-GM–based oral cavity cancer classifications commonly categorize malignant neoplasms of the lip within the broader oral cavity cancer spectrum.

Only hospitalizations with an overnight stay were eligible for inclusion. Hospitalizations were identified exclusively based on ICD-10-GM diagnosis coding. Recurrent tumors, metastatic disease, and secondary primary tumors could not be separately identified within the administrative dataset. Diagnoses were captured at the four- to five-digit ICD-10-GM level, and the exact subcode distribution is provided in Supplementary Table S7. Because selected subsites within these categories anatomically belong to the oropharynx rather than the oral cavity (base of tongue, C01; lingual tonsil, C02.4; soft palate, C05.1; and uvula, C05.2), a sensitivity analysis restricted to unambiguous oral cavity subsites was performed.

### 2.3. Study Outcome and Variables

The primary outcome was hospital LOS, measured in days. In addition to reporting median LOS, we assessed the proportion of hospitalizations with prolonged stay, defined as LOS ≥ 7 days and LOS ≥ 14 days. These thresholds were selected empirically based on the LOS distribution within the study population. LOS ≥ 7 days represented hospitalization durations exceeding the median LOS of the cohort (median: 6 days), whereas LOS ≥ 14 days represented hospitalization durations exceeding the 75th percentile of the LOS distribution (75th percentile: 13 days).

Several variables were included to characterize the study population and to evaluate their association with LOS. Demographic variables comprised mean age, predefined age categories (≤60, 61–70, 71–80, and >80 years), and sex.

Multimorbidity was assessed using the van Walraven weighted Elixhauser Comorbidity Score, which assigns weighted values (ranging from −7 to +12) to 30 comorbidity categories (e.g., congestive heart failure, cardiac arrhythmias, valvular disease, pulmonary circulation disorders, peripheral vascular disease, uncomplicated and complicated hypertension, paralysis, other neurological disorders, chronic pulmonary disease, uncomplicated and complicated diabetes, hypothyroidism, renal failure, liver disease, peptic ulcer disease, HIV/AIDS, lymphoma, metastatic cancer, solid tumors without metastasis, rheumatoid arthritis/collagen vascular disease, coagulopathy, obesity, weight loss, fluid and electrolyte disorders, blood loss anemia, deficiency anemia, alcohol abuse, drug abuse, psychoses, and depression) [12]. The score was categorized into low (<5), moderate 5–14), and high (>14) comorbidity burden.

Furthermore, procedure-related variables were defined based on the German Operation and Procedure Classification System (OPS), derived from the German modification of the International Classification of Procedures in Medicine (ICPM). These included chemotherapy (OPS: 8-542, 8-543, 8-544), radiotherapy (OPS: 8-52), surgical procedures in the oral and maxillofacial region (OPS: 5-25, 5-26, 5-27), lymphatic system operations (OPS: 5-40), including cervical lymph node and regional neck dissection procedures. To address potential procedural overlap between oral and facial surgery and lymphatic system operations, an additional sensitivity analysis was performed in which these two procedure variables were replaced by a four-category surgical variable: (0) no surgery (reference), (1) oral and facial surgery only, (2) lymphatic system operations only, and (3) both procedures combined., transfusion of blood cells (OPS: 8-800), and complex intensive care treatment (OPS: 8-980, 8-98f, 8-98d).

### 2.4. Statistical Analysis

Associations between demographic and clinical variables and hospital LOS were examined using multivariable Poisson regression models. Results are presented as rate ratios (RRs) with corresponding 95% confidence intervals (CIs) and *p*-values. To account for potential inter-hospital variability in patient profiles, clinical practices, and resource availability, hospital was included as a random intercept in all multivariable regression models using generalized linear mixed models (PROC GLIMMIX, SAS 9.4). The Poisson model was estimated using Laplace approximation (method = Laplace). The logistic regression models were estimated using residual pseudo-likelihood. For all three models, the statistical significance of the random intercept variance was assessed using a Wald test.

Associations between demographic and clinical variables and prolonged LOS were analyzed using multivariable logistic regression models, with results reported as odds ratios (ORs), 95% CIs, and *p*-values.

All models were adjusted for cancer type, age, sex, van Walraven-weighted Elixhauser Comorbidity Score, and procedure-related variables. A two-sided significance level of *p* < 0.05 was applied. To improve transparency and facilitate assessment of the magnitude of confounding, univariable (unadjusted) Poisson and logistic regression analyses were additionally performed. To provide a more detailed characterization of surgical procedures, an additional sensitivity analysis replaced oral and facial surgery and lymphatic system operations with a combined four-category surgical variable (no surgery, oral and facial surgery only, lymphatic system operations only, or both combined). To characterize treatment modalities in more detail, a further sensitivity analysis replaced radiotherapy and chemotherapy with a five-category treatment variable: no systemic therapy (reference), radiotherapy only, chemotherapy only, combined radiotherapy and chemotherapy, or immunotherapy and targeted therapy (OPS: 6-002). Immunotherapy was assigned as the priority category; patients receiving immunotherapy concurrently with other modalities were classified as immunotherapy.

All statistical analyses were performed using SAS version 9.4 (SAS Institute, Cary, NC, USA).

Hospital length of stay was additionally summarized by its mean and standard deviation. Model diagnostics indicated overdispersion of the count outcome (Pearson χ^2^/df = 6.7 in the marginal Poisson model). The Poisson model was retained as the primary model for its direct interpretability, and a negative binomial mixed model with the same covariates and hospital random intercept was fitted as a sensitivity analysis to assess the stability of the associations (Supplementary Table S9). As a further sensitivity analysis addressing the observed overdispersion, a robust (sandwich-variance) Poisson model with standard errors clustered by hospital was estimated using generalized estimating equations with a working-independence correlation structure (Supplementary Table S10).

To address the anatomical overlap between the oral cavity and the oropharynx within ICD-10-GM codes C00–C06, a further sensitivity analysis was restricted to unambiguous oral cavity subsites by excluding hospitalizations coded for base of tongue (C01), lingual tonsil (C02.4), soft palate (C05.1), or uvula (C05.2) (Supplementary Table S8).

All analyses were complete-case analyses; no missing data were present for any of the analyzed variables.

### 2.5. Ethics and Data Governance

The analysis used fully anonymized inpatient administrative records containing no directly or indirectly identifiable patient data. Prior to provision for research, all records were anonymized in accordance with applicable data-protection regulations. Under the EU General Data Protection Regulation (GDPR, Recital 26) and the German Federal Data Protection Act (Bundesdatenschutzgesetz, BDSG §27), research using anonymized data does not involve identifiable individuals and therefore requires neither ethics-committee approval nor informed consent; this also constituted the legal basis for the secondary analysis of these administrative data.

## 3. Results

### 3.1. Baseline Characteristics

A total of 3957 inpatient hospitalizations for OCC were included. These hospitalizations originated from 34 of the 49 hospitals included in the database. Thirteen of these hospitals contributed more than 10 OCC hospitalizations each, with a median of 196 OCC hospitalizations among these higher-volume centers (interquartile range [IQR]: 51–307). Mean age was 65.6 years (SD 11.3). Overall, 32.9% of hospitalizations involved patients aged ≤60 years, 36.1% involved patients aged 61–70 years, 20.0% involved patients aged 71–80 years, and 11.0% involved patients aged >80 years. Male patients accounted for 66.2% of hospitalizations and female patients for 33.8%.

Regarding cancer site category, tumors of the tongue were most frequent (50.7%), followed by floor of mouth (20.4%), gum (10.5%), palate (8.0%), other and unspecified parts of the mouth (6.4%), and lip (4.0%). Based on the van Walraven weighted Elixhauser Score, 40.3% of hospitalizations were classified as having a low comorbidity burden, 25.9% as moderate, and 33.8% as high.

Among procedure-related variables, surgical procedures in the oral and facial region were recorded in 32.2% of hospitalizations, lymphatic system operations in 25.1%, radiotherapy in 21.8%, chemotherapy in 15.7%, transfusion of blood cells in 11.8%, and complex intensive care treatment in 13.6% (Table 1).

### 3.2. Median Hospital Length of Stay and Prolonged Hospitalization

The median LOS in the total study population was 6 days (IQR 3–13). The mean LOS was 10.2 days (standard deviation 11.8). Overall, 49.4% of hospitalizations had an LOS ≥ 7 days and 23.5% had an LOS ≥ 14 days.

Median LOS increased with age, ranging from 6 days in hospitalizations involving patients aged ≤60 years to 7 days in those involving patients aged >70 years. The proportion of prolonged hospitalization was highest among hospitalizations involving patients aged >80 years (57.0% for LOS ≥ 7 days; 27.0% for LOS ≥ 14 days).

LOS also differed across cancer site categories. Hospitalizations related to gum cancer and to other or unspecified parts of the mouth showed higher median LOS (7 and 8 days, respectively) than hospitalizations related to lip and tongue cancer (6 days each). The highest proportion of LOS ≥ 14 days was observed in gum cancer (32.6%).

A clear gradient was observed according to comorbidity burden. Median LOS was 5 days (IQR 3–8) in hospitalizations with low comorbidity burden, compared with 8 days (IQR 4–15) in those with moderate burden and 8 days (IQR 4–18) in those with high burden. The proportion of LOS ≥ 14 days increased from 9.9% in the low-comorbidity group to 34.6% in the high-comorbidity group.

Procedure-related differences were substantial. Hospitalizations involving transfusion of blood cells or complex intensive care treatment showed markedly longer median LOS (20 and 18 days, respectively) and the highest proportions of LOS ≥ 14 days (68.6% and 69.7%, respectively) (Table 2).

Median LOS across participating hospitals is additionally shown in Supplementary Figure S1.

### 3.3. Association of Demographic and Clinical Variables with Hospital Length of Stay

Univariable (unadjusted) Poisson regression results are presented in Supplementary Table S1. Comparing crude and adjusted estimates allowed assessment of confounding; for most variables, adjusted estimates were attenuated relative to unadjusted estimates, consistent with mutual confounding among age, comorbidity, and treatment-related variables. The random intercept for hospital was statistically significant in all three multivariable models (Poisson: variance estimate 0.098, *p* = 0.002; logistic LOS ≥ 7 days: variance estimate 0.311, *p* = 0.022; logistic LOS ≥ 14 days: variance estimate 0.258, *p* = 0.031), indicating meaningful inter-hospital variability that was appropriately accounted for in the analyses. In multivariable Poisson regression models with hospital as a random intercept, age 71–80 years was not significantly associated with LOS (RR 1.03; 95% CI 1.00–1.06; *p* = 0.065), while age > 80 years was associated with a longer LOS (RR 1.17; 95% CI 1.13–1.21; *p* < 0.001). Age 61–70 years was not significantly associated with LOS (RR 1.00; 95% CI 0.98–1.03; *p* = 0.788).

Male sex was associated with a longer LOS than female sex (RR 1.09; 95% CI 1.07–1.12; *p* < 0.001).

Compared with tongue cancer, lip cancer was associated with a shorter LOS (RR 0.88; 95% CI 0.83–0.94; *p* < 0.001). Gum cancer (RR 1.10; 95% CI 1.06–1.13; *p* < 0.001), floor-of-mouth cancer (RR 1.06; 95% CI 1.03–1.09; *p* < 0.001), and palate cancer (RR 1.15; 95% CI 1.10–1.19; *p* < 0.001) were associated with longer LOS. Tumors of other and unspecified parts of the mouth were not significantly associated with LOS (RR 0.99; 95% CI 0.95–1.03; *p* = 0.615).

A strong association was observed for comorbidity burden. Compared with low comorbidity burden, moderate comorbidity burden was associated with longer LOS (RR 1.38; 95% CI 1.34–1.42; *p* < 0.001), and high comorbidity burden was associated with even longer LOS (RR 1.58; 95% CI 1.54–1.63; *p* < 0.001).

**Figure 1:**
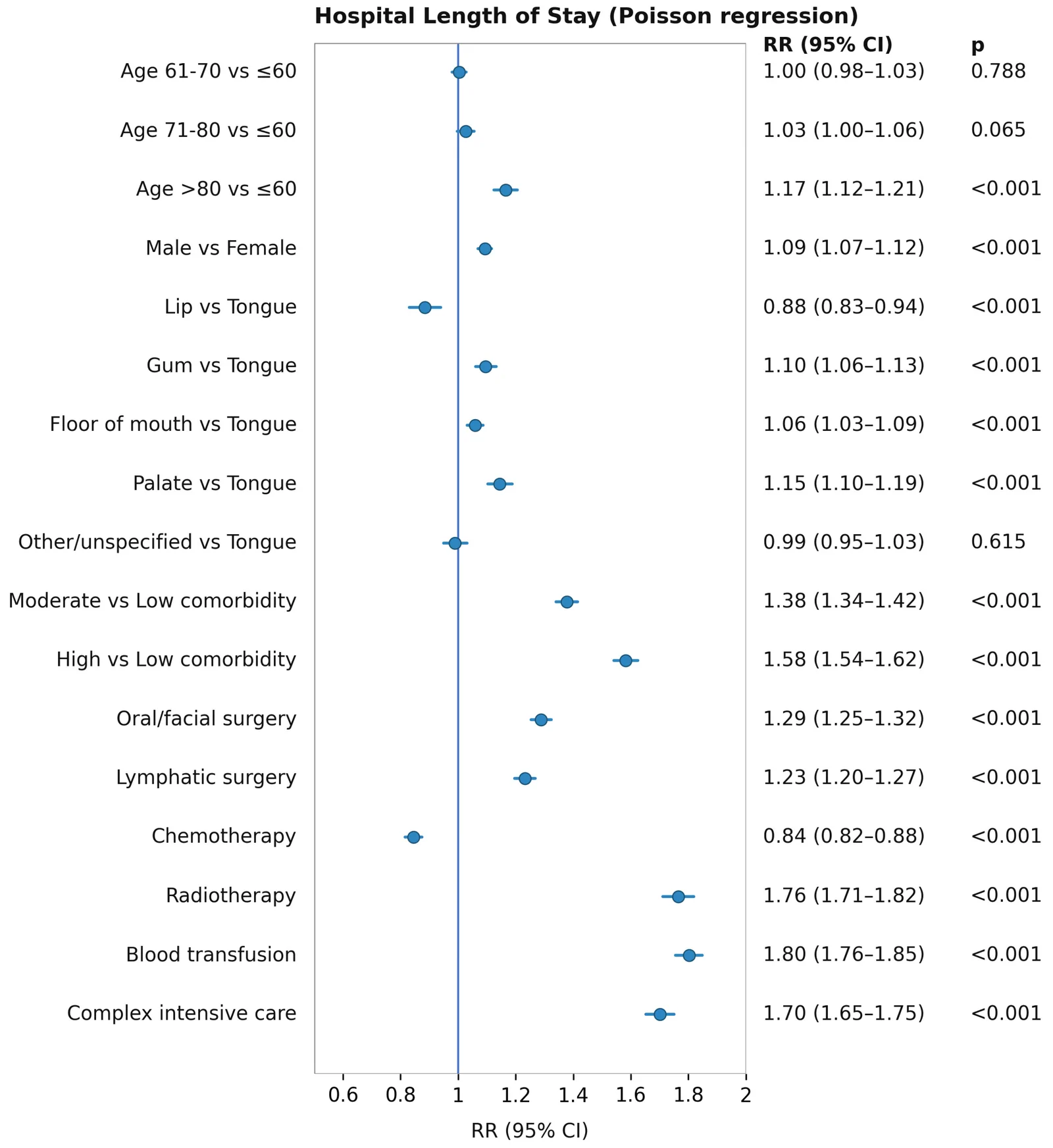
Multivariable Poisson regression analysis of demographic, clinical, and treatment-related variables associated with hospital length of stay in inpatient hospitalizations for oral cavity cancer (hospital included as a random intercept). Reference categories: age ≤ 60 years; female sex; tongue cancer; low comorbidity burden; no respective procedure for all procedure-related variables.

Among treatment-related variables, compared with hospitalizations without the respective procedure, surgical procedures in the oral and facial region (RR 1.29; 95% CI 1.25–1.32; *p* < 0.001), lymphatic system operations (RR 1.23; 95% CI 1.20–1.27; *p* < 0.001), radiotherapy (RR 1.77; 95% CI 1.71–1.82; *p* < 0.001), transfusion of blood cells (RR 1.80; 95% CI 1.76–1.85; *p* < 0.001), and complex intensive care treatment (RR 1.70; 95% CI 1.65–1.75; *p* < 0.001) were associated with longer LOS. Chemotherapy was associated with shorter LOS (RR 0.85; 95% CI 0.82–0.88; *p* < 0.001) (Figure 1).

### 3.4. Association of Demographic and Clinical Variables with Prolonged Hospitalization

Results of the corresponding univariable logistic regression analyses are presented in Supplementary Table S2. In multivariable logistic regression analyses, both LOS ≥ 7 days and LOS ≥ 14 days were consistently associated with older age, higher comorbidity burden, and markers of more complex inpatient treatment. For LOS ≥ 7 days, age > 80 years was associated with higher odds of prolonged hospitalization (OR 1.58; 95% CI 1.20–2.07; *p* = 0.001), whereas the other age categories were not significantly associated. Sex was not significantly associated with LOS ≥ 7 days. No statistically significant associations between cancer site category and LOS ≥ 7 days were observed. Moderate comorbidity burden (OR 2.12; 95% CI 1.75–2.57; *p* < 0.001) and high comorbidity burden (OR 2.42; 95% CI 2.00–2.93; *p* < 0.001) were associated with increased odds of LOS ≥ 7 days. Compared with hospitalizations without the respective procedure, surgical procedures in the oral and facial region (OR 1.91; 95% CI 1.56–2.32; *p* < 0.001), lymphatic system operations (OR 3.25; 95% CI 2.61–4.06; *p* < 0.001), radiotherapy (OR 2.42; 95% CI 1.91–3.06; *p* < 0.001), transfusion of blood cells (OR 4.80; 95% CI 3.49–6.62; *p* < 0.001), and complex intensive care treatment (OR 10.63; 95% CI 6.74–16.78; *p* < 0.001) were associated with increased odds of LOS ≥ 7 days, whereas chemotherapy was associated with lower odds (OR 0.36; 95% CI 0.28–0.47; *p* < 0.001). Corresponding unadjusted estimates are presented in Supplementary Table S2.

**Figure 2:**
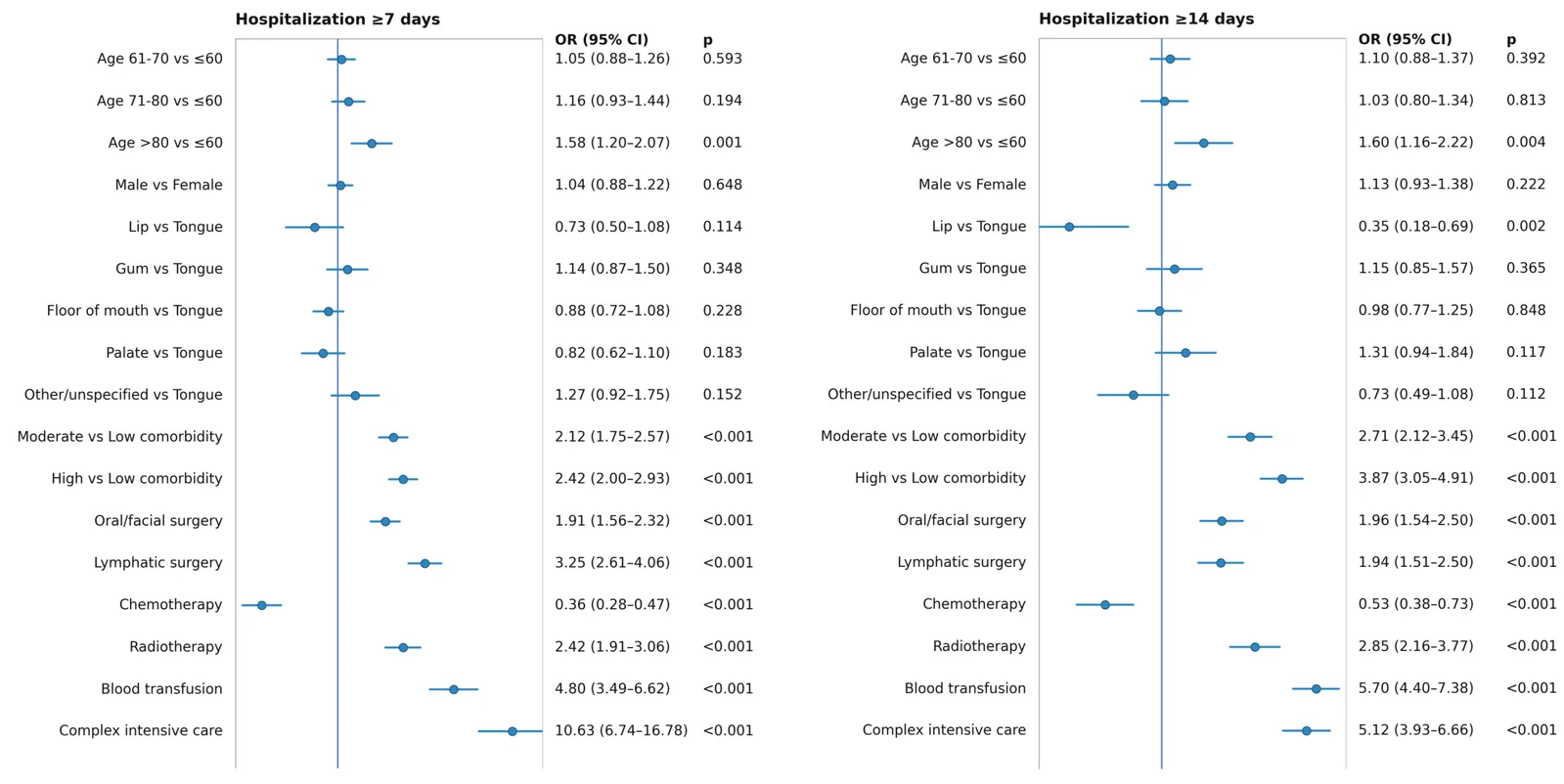
Multivariable logistic regression analysis of demographic, clinical, and treatment-related variables associated with prolonged hospitalization (LOS ≥ 7 days and LOS ≥ 14 days) in inpatient hospitalizations for oral cavity cancer (hospital included as random intercept). Reference categories: age ≤ 60 years; female sex; tongue cancer; low comorbidity burden; no respective procedure for all procedure-related variables.

For LOS ≥ 14 days, age > 80 years was associated with increased odds (OR 1.61; 95% CI 1.16–2.22; *p* = 0.004). Lip cancer was associated with lower odds (OR 0.35; 95% CI 0.18–0.69; *p* = 0.002), whereas the other cancer site categories were not significantly associated. Moderate comorbidity burden (OR 2.71; 95% CI 2.12–3.45; *p* < 0.001) and high comorbidity burden (OR 3.87; 95% CI 3.05–4.91; *p* < 0.001) were associated with increased odds of LOS ≥ 14 days. Compared with hospitalizations without the respective procedure, surgical procedures in the oral and facial region (OR 1.96; 95% CI 1.54–2.50; *p* < 0.001), lymphatic system operations (OR 1.94; 95% CI 1.51–2.50; *p* < 0.001), radiotherapy (OR 2.85; 95% CI 2.16–3.77; *p* < 0.001), transfusion of blood cells (OR 5.70; 95% CI 4.40–7.38; *p* < 0.001), and complex intensive care treatment (OR 5.12; 95% CI 3.93–6.66; *p* < 0.001) were associated with increased odds of LOS ≥ 14 days, whereas chemotherapy was associated with lower odds (OR 0.53; 95% CI 0.38–0.73; *p* < 0.001) (Figure 2). Notably, some associations differed between the LOS ≥ 7 days and LOS ≥ 14 days analyses. In particular, palate cancer and other or unspecified parts of the mouth showed a shift in the direction or magnitude of association between the two cutoffs. In the unadjusted analyses (Supplementary Table S2), palate cancer was not associated with either LOS ≥ 7 days (OR 0.99; 95% CI 0.78–1.26) or LOS ≥ 14 days (OR 1.24; 95% CI 0.64–1.63), and other or unspecified parts of the mouth showed a positive association with LOS ≥ 7 days (OR 1.51; 95% CI 1.16–1.96) but not with LOS ≥ 14 days (OR 1.04; 95% CI 0.76–1.43). These variations likely reflect differences in subgroup size, distributional characteristics across LOS thresholds, and residual confounding, and should be interpreted with caution. Age- and sex-adjusted analyses of individual Elixhauser comorbidity components with hospital random intercept are presented in Supplementary Table S3. Coagulopathy showed the strongest individual association with longer LOS (RR 2.91), followed by fluid and electrolyte disorders (RR 2.18) and congestive heart failure (RR 1.97). A sensitivity analysis excluding lip cancer (C00; remaining codes C01–C06, n = 3800) yielded highly consistent results across all three models (Supplementary Table S4). A sensitivity analysis using a combined four-category surgical variable (no surgery, oral and facial surgery only, lymphatic system operations only, both combined) demonstrated that hospitalizations with both procedures combined were most strongly associated with prolonged LOS, particularly for LOS ≥ 7 days (OR 9.59; 95% CI 7.23–12.72; *p* < 0.001) (Supplementary Table S5). A sensitivity analysis using a five-category treatment variable demonstrated that radiotherapy alone was most strongly associated with longer LOS (RR 1.75; OR 2.08 for LOS ≥ 7 days; OR 2.52 for LOS ≥ 14 days), whereas combined radiotherapy and chemotherapy showed a paradoxically lower OR for LOS ≥ 7 days (OR 0.67; 95% CI 0.52–0.86). Immunotherapy and targeted therapy (n = 82, 2.1%) were associated with longer LOS by Poisson regression (RR 1.89; 95% CI 1.80–1.99) but not significantly with binary LOS outcomes (Supplementary Table S6).

Overall, prolonged LOS was predominantly associated with advanced age, higher comorbidity burden, and indicators of more complex inpatient treatment.

The exact ICD-10-GM subcode distribution is shown in Supplementary Table S7; codes anatomically attributable to the oropharynx accounted for 996 hospitalizations (25.2%), predominantly base of tongue (C01, 20.9% of coded diagnoses). A sensitivity analysis restricted to unambiguous oral cavity subsites (n = 2961) yielded associations consistent in direction, magnitude, and significance with the main analysis for comorbidity burden (high burden RR 1.73, 95% CI 1.68–1.79), age > 80 years (RR 1.16, 95% CI 1.11–1.20), blood transfusion, complex intensive care, surgical procedures, and radiotherapy, and confirmed the inverse association with chemotherapy (RR 0.91, 95% CI 0.87–0.95; Supplementary Table S8). Model diagnostics indicated overdispersion (marginal Pearson χ^2^/df = 6.7); a negative binomial mixed model accounting for overdispersion showed good conditional fit (Pearson χ^2^/df = 1.33) and yielded associations consistent in direction and significance with the Poisson model, with slightly wider confidence intervals (Supplementary Table S9). A robust Poisson model with hospital-clustered sandwich standard errors yielded rate ratios consistent with the main analysis; all principal associations—comorbidity burden, age > 80 years, blood transfusion, complex intensive care, surgical procedures, radiotherapy, and the inverse chemotherapy association—remained statistically significant despite the wider, overdispersion- and cluster-robust confidence intervals (Supplementary Table S10).

## 4. Discussion

In this multicenter analysis of adult inpatient hospitalizations for OCC in Germany, we found that LOS varied substantially across hospitalizations, with a median LOS of 6 days, nearly half of all stays lasting at least 7 days, and approximately one quarter extending to two weeks or longer. Older age, higher comorbidity burden, and multiple treatment-related variables indicative of complex inpatient management were consistently associated with longer hospital stay and prolonged LOS. These findings support LOS as an integrative marker of treatment intensity, patient vulnerability, and perioperative care complexity in OCC. The observed LOS distribution is broadly consistent with previous findings from head and neck oncology, in which frailty, multimorbidity, postoperative complications, extensive surgery, and intensive care requirements have been associated with prolonged hospitalization [5,6,7,8,9,11,13,14]. However, the median LOS in our cohort was lower than reported in several international studies, including population-based and surgical series describing mean postoperative stays of approximately 10 days or longer [5,6,8]. Such cross-study comparisons should be interpreted with caution, as prior studies often focused on highly selected surgical populations, whereas the present analysis reflects a broader set of inpatient hospitalizations captured in routine administrative data and captures a full range of treatment pathways within contemporary OCC care [5,6,8,9].

Several previous studies provide clinically relevant context for our findings. Lindeborg et al. analyzed 282 patients undergoing head and neck oncologic resection with free flap reconstruction and reported a median LOS of 10 days; factors associated with longer LOS included return to the operating room for flap-related complications, pneumonia or urinary tract infection, wound breakdown or fistula, surgical site infection, and prior radiotherapy [5]. Similarly, McDevitt et al. linked cancer registry and hospital discharge data in Ireland and found that tracheostomy, postoperative infection, neoadjuvant radiotherapy, advanced stage, gastrostomy, and reconstruction were associated with longer hospitalization after head and neck cancer surgery [6]. Denholm et al. focused specifically on oral cancer patients undergoing free flap reconstruction and identified increasing age, elective tracheostomy, floor-of-mouth or mandibular tumors, longer operating time, and fibular free flap reconstruction as determinants of extended postoperative stay [8]. These findings are consistent with our observation that surgical procedures, lymphatic system operations, radiotherapy, transfusion of blood cells, and complex intensive care treatment were associated with longer LOS, although our administrative dataset did not contain detailed information on tracheostomy, flap type, operating time, postoperative infections, or wound complications.

Further support comes from studies focusing on intensive care, frailty, and comorbidity. Aponte-Ortiz et al. reported that postoperative ICU management after head and neck free flap reconstruction was associated with longer hospitalization and higher short-term costs without clear improvement in major postoperative outcomes [7]. Nieman et al. showed in a large inpatient sample that frailty was associated with postoperative complications, increased LOS, and higher costs after head and neck cancer surgery [11]. Genther and Gourin similarly reported that advanced comorbidity in elderly patients undergoing head and neck cancer surgery was associated with increased mortality, morbidity, length of hospitalization, and costs [13]. Raab et al. further demonstrated that geriatric assessment-defined frailty was associated with longer postoperative hospitalization and ICU admission among older adults with head and neck cancer [14]. In line with these studies, our analysis showed that advanced age and higher van Walraven weighted Elixhauser comorbidity burden were independently associated with longer LOS and with both LOS ≥ 7 days and LOS ≥ 14 days.

In addition, LOS may indirectly reflect underlying tumor burden and disease extent, as advanced-stage tumors frequently require more extensive surgery, reconstruction, perioperative monitoring, and supportive care. Consequently, the absence of TNM and UICC stage information represents an important limitation of the present analysis, because disease stage is likely a major determinant of treatment complexity and hospitalization burden in OCC. However, detailed tumor staging variables were unavailable in the present administrative dataset. Structural characteristics of the German hospital system may further contribute to the comparatively shorter LOS in this analysis. In Germany, hospital reimbursement is largely based on diagnosis-related groups (DRGs), which may incentivize efficient inpatient workflows and streamlined perioperative pathways [17]. Nevertheless, existing evidence suggests that DRG-based reimbursement does not uniformly result in shorter hospital stays, indicating that LOS arises from a complex interplay between system-level influences and patient- and treatment-related determinants rather than from reimbursement mechanisms alone [17].

Advanced age and comorbidity burden emerged as central correlates of prolonged LOS and may be understood as overlapping dimensions of patient vulnerability. Hospitalizations involving patients aged >80 years and those with moderate to high comorbidity burden are consistently associated with longer stays. The observation aligns with prior literature demonstrating that older adults with head and neck cancer often experience greater postoperative morbidity, longer hospitalizations, and higher care complexity [11,13,14]. Similar associations between frailty, comorbidity burden, postoperative complications, and extended hospitalization have also been reported across broader oncological and surgical populations, emphasizing the importance of biological vulnerability beyond chronological age alone [18,19,20,21]. Recent studies have further highlighted frailty, sarcopenia, and malnutrition risk as important determinants of adverse postoperative outcomes, delayed recovery, and increased care complexity in patients with head and neck cancer [22,23,24,25,26].

Our study supports the notion that multimorbidity substantially amplifies inpatient care complexity in OCC and may be particularly relevant for perioperative risk stratification and discharge planning [27,28]. The present analysis focused on overall multimorbidity burden as summarized by the van Walraven weighted Elixhauser Comorbidity Score. To further explore which individual comorbidity components contribute most prominently to prolonged hospitalization, age- and sex-adjusted Poisson regression analyses were performed for each Elixhauser comorbidity separately, with hospital included as a random intercept. Coagulopathy showed the strongest association with longer LOS (RR 2.91; 95% CI 2.78–3.03), followed by fluid and electrolyte disorders (RR 2.18; 95% CI 2.13–2.22), congestive heart failure (RR 1.97; 95% CI 1.90–2.04), valvular disease (RR 1.95; 95% CI 1.84–2.07), and pulmonary circulation disorders (RR 1.88; 95% CI 1.76–2.00). Weight loss was also strongly associated with longer LOS (RR 1.86; 95% CI 1.80–1.92), likely reflecting nutritional depletion and frailty in this population. Notably, coagulopathy showed the highest rate ratio despite its relatively low van Walraven weight of 3, suggesting that its contribution to hospitalization burden in OCC may exceed its weight in the global comorbidity score. These findings refine the interpretation of the overall comorbidity burden and highlight specific comorbidity entities that may be particularly relevant for perioperative risk stratification in OCC.

Treatment-related variables were also strongly associated with LOS, including surgical procedures involving the oral and facial region, lymphatic system operations, transfusion of blood cells, and complex intensive care treatment. These associations are clinically plausible and consistent with prior literature linking extensive surgery, free flap reconstruction, tracheostomy, infectious complications, and intensive care treatment to prolonged hospitalization in head and neck cancer [5,6,7,8,9].

Because OPS-based administrative coding does not reliably distinguish isolated lymphatic procedures from neck dissections performed concurrently with primary tumor surgery, we performed an additional sensitivity analysis using a combined four-category surgical variable: no surgery (reference), oral and facial surgery only, lymphatic system operations only, and both procedures combined (Supplementary Table S5). This analysis revealed that hospitalizations involving both surgical procedures simultaneously showed substantially stronger associations with prolonged LOS than either procedure alone, particularly for LOS ≥ 7 days (OR 9.59; 95% CI 7.23–12.72), consistent with these representing the most complex surgical cases. Hospitalizations with oral and facial surgery only (OR 1.20; 95% CI 0.95–1.51, *p* = 0.130) and lymphatic operations only (OR 1.24; 95% CI 0.90–1.70, *p* = 0.191) were not independently associated with LOS ≥ 7 days, while both showed associations with LOS ≥ 14 days. These findings confirm that the separate procedure variables in the main model reflect distinct and complementary aspects of surgical complexity rather than redundant coding of the same intervention. Furthermore, in the context of hospitalization-level administrative data, these variables should not be interpreted as baseline predictors or direct causal determinants present at admission. Rather, they likely reflect treatment intensity, perioperative complexity, and complicated inpatient courses that co-occur with prolonged LOS. Because prolonged LOS was common (LOS ≥ 7 days in 49.4% of hospitalizations), the odds ratios from the logistic models exceed the corresponding relative risks; the odds ratio for complex intensive care (approximately 10.6 for LOS ≥ 7 days) should therefore not be interpreted as a tenfold increase in risk.

Chemotherapy was associated with shorter LOS, a finding that may reflect more standardized and time-limited inpatient treatment episodes compared with surgery-driven hospitalizations. Radiotherapy was consistently associated with prolonged LOS across both descriptive and adjusted analyses. This finding is in line with previous reports from head and neck cancer cohorts, in which hospitalization during radiotherapy was a frequent event reflecting acute treatment toxicity and was associated with poorer survival [29], and in which a higher mean radiation dose to the oral cavity predicted opioid requirement and hospitalization during treatment [30]. To provide a more detailed characterization of treatment modalities, a sensitivity analysis was performed using a five-category treatment variable (no systemic therapy, radiotherapy only, chemotherapy only, combined radiotherapy and chemotherapy, immunotherapy/targeted therapy; Supplementary Table S6). Radiotherapy alone was associated with longer LOS (RR 1.75; 95% CI 1.69–1.81; OR 2.08 for LOS ≥ 7 days; OR 2.52 for LOS ≥ 14 days). Combined radiotherapy and chemotherapy (n = 503) was associated with longer LOS in the Poisson model (RR 1.48) but with lower odds of LOS ≥ 7 days (OR 0.67; 95% CI 0.52–0.86), likely reflecting predominantly short-stay concurrent chemoradiotherapy episodes. Chemotherapy alone (n = 72) showed a borderline association with longer LOS (RR 1.08; *p* = 0.050). Immunotherapy and targeted therapy (OPS 6-002; n = 82, 2.1%) were associated with longer LOS in the Poisson model (RR 1.89; 95% CI 1.80–1.99) but not significantly with LOS ≥ 7 days or ≥14 days, likely reflecting the small sample size and wide confidence intervals. These agents, including the anti-EGFR monoclonal antibody cetuximab and the PD-1 immune checkpoint inhibitors nivolumab and pembrolizumab, have demonstrated survival benefit in locoregionally advanced and recurrent or metastatic head and neck squamous cell carcinoma [31,32]; their presence in this administrative dataset, while limited in number, confirms their use in routine clinical care during the study period. These findings should therefore be interpreted cautiously and primarily as indicators of differing treatment contexts rather than as direct effects on hospitalization duration. In addition, some associations differed between the LOS ≥ 7 days and LOS ≥ 14 days analyses, particularly for selected cancer site categories such as palate cancer and other or unspecified parts of the mouth. These variations may reflect differences in subgroup size, distributional characteristics, and residual confounding, emphasizing the importance of cautious interpretation of threshold-based LOS analyses.

From a translational and health services perspective, these findings may support development of inpatient care pathways and resource planning, optimization of discharge planning, and improved allocation of inpatient resources for patients with OCC. In addition, the study highlights the value of real-world administrative datasets for identifying hospitalization patterns and vulnerable patient populations in routine oncological care. From a nursing and care-coordination perspective, and as practice implications rather than measured outcomes, these associations may inform discharge-readiness assessment, nutritional and swallowing support, tracheostomy-related and mobility care, caregiver preparation, and multidisciplinary discharge coordination.

### Strengths and Limitations

This study has several strengths. It used a multicenter inpatient database, drew on a source database spanning different hospital types, and reflects routine care under real-world conditions in participating German hospitals. Moreover, the analysis simultaneously considered demographic variables, tumor site categories, comorbidity burden, and treatment-related variables in a relatively large cohort of inpatient hospitalizations. Hospital was included as a random intercept in all multivariable models to account for inter-institutional variability. Several sensitivity analyses were performed to address methodological concerns, including a sensitivity analysis excluding lip cancer (C00), a combined surgical category analysis, a disaggregated treatment modality analysis including immunotherapy, and a component-level comorbidity analysis. Nevertheless, several limitations must be acknowledged. First, the study was based on administrative case data and therefore lacked detailed clinical information such as TNM classification, UICC stage, histopathological subtype at the individual patient level, molecular characteristics, recurrence status, functional status, frailty measures, smoking and alcohol history, and exact treatment intent. The absence of TNM and UICC staging data represents a key limitation, as disease stage is likely a major determinant of treatment complexity and hospitalization duration in OCC. Although OPS-based treatment categories allowed identification of chemotherapy, radiotherapy, immunotherapy, and targeted therapy procedures, exact therapeutic agents, treatment protocols, dosing schedules, and treatment sequencing were not consistently available. To partially address these limitations, several sensitivity analyses were performed, including a sensitivity analysis excluding lip cancer (C00), a combined surgical category analysis, and a disaggregated treatment modality analysis including immunotherapy. These analyses were used to assess the stability of the principal associations, which were consistent across the alternative specifications, despite the inherent constraints of administrative data.

Because the administrative database relied primarily on ICD-10-GM diagnosis coding, the cohort included different malignant oral cavity tumor subtypes; however, OSCC likely represented the predominant entity, and a sensitivity analysis restricted to C01–C06 demonstrated robustness of all main associations. Second, because the data were anonymized and analyzed at the hospitalization level, recurrent admissions of the same individual, if present, were counted as separate inpatient episodes. Accordingly, the findings should be interpreted as applying to hospitalizations rather than to unique patients. Third, inter-hospital variability in coding practices and patient profiles cannot be fully excluded despite the inclusion of hospital as a random intercept in all multivariable models; the random intercept was statistically significant in all three models, confirming that this source of variability was appropriately accounted for in the analyses. Fourth, although the source database covered 49 hospitals, OCC-related hospitalizations originated from 34 centers (13 of which contributed more than 10 OCC hospitalizations each), and the care level and type of these contributing hospitals were not available; representativeness for German OCC inpatient care therefore cannot be assured, and selection bias cannot be excluded. Fifth, the use of ICD-based administrative definitions may not fully capture all clinically nuanced oral cavity subsites. Finally, because of the observational cross-sectional design, the findings should be interpreted as associations rather than causal effects. In addition, ICD-10-GM codes C00–C06 include subsites that anatomically belong to the oropharynx (notably base of tongue, C01); a sensitivity analysis restricted to unambiguous oral cavity subsites confirmed the robustness of the principal associations. Finally, because the data were anonymized, repeat admissions by the same patient could not be linked, and potential within-patient clustering could not be accounted for.

## 5. Conclusions

In this multicenter study of adult inpatient hospitalizations for OCC in Germany, the median LOS was 6 days. Older age, higher comorbidity burden, and several treatment-related markers of more complex inpatient management were associated with extended hospital stay, whereas chemotherapy-related hospitalizations were associated with shorter LOS. Overall, our results position LOS as a clinically meaningful surrogate for treatment burden and patient vulnerability in OCC. Rather than a causal determinant, LOS was associated with comorbidity burden, advanced age, and markers of inpatient treatment and clinical complexity. In addition, our findings highlight the importance of improving the completeness and standardization of hospital documentation systems. More consistent recording of oncological characteristics, treatment trajectories, complications, and longitudinal patient identifiers in administrative databases may improve future real-world analyses and facilitate more precise evaluation of inpatient outcomes in OCC. From a health services perspective, these insights may inform hospital benchmarking, resource allocation, and the development of inpatient resource planning, discharge coordination, and multidisciplinary inpatient care pathways in OCC management.

## Figures and Tables

**Table 1:** Baseline characteristics of the study sample. Procedure categories are not mutually exclusive; a single hospitalization may contribute to more than one procedure category.

Variable	N (%), Mean (SD) or Median (IQR) (n = 3957)
Age	
Mean age (SD)	65.6 (11.3)
≤60 years, n (%)	1302 (32.9)
61–70 years, n (%)	1428 (36.1)
71–80 years, n (%)	790 (20.0)
>80 years, n (%)	437 (11.0)
Sex, n (%)	
Female	1338 (33.8)
Male	2619 (66.2)
Primary cancer diagnosis (n, %)	
Lip	157 (4.0)
Tongue	2006 (50.7)
Gum	417 (10.5)
Floor of mouth	808 (20.4)
Palate	316 (8.0)
Other and unspecific parts of mouth	253 (6.4)
van Walraven weighted Elixhauser Score (n, %)	
Low	1594 (40.3)
Moderate	1024 (25.9)
High	1339 (33.8)
Procedures (n, %)	
Surgical procedures in the oral and facial region	1273 (32.2)
Operations on the lymphatic system	994 (25.1)
Radiotherapy	863 (21.8)
Chemotherapy	620 (15.7)
Transfusion of blood cells	465 (11.8)
Complex intensive care	537 (13.6)

**Table 2:** Median hospital length of stay (days) and proportion of hospitalizations with prolonged length of stay in patients with oral cavity cancers. Values are reported at the hospitalization level; procedure categories are not mutually exclusive.

Patient Group	Median (IQR)	≥7 Days (%)	≥14 Days (%)
Total	6 (3–13)	49.4	23.5
Age			
Age ≤ 60 years	6 (3–12)	47.9	22.1
Age 61–70 years	6 (3–13)	47.2	22.6
Age 71–80 years	7 (4–14)	51.8	25.3
Age > 80 years	7 (3–14)	57.0	27.0
Sex			
Female	6 (3–13)	49.9	23.9
Male	6 (3–13)	49.1	23.3
Primary cancer diagnosis			
Lip	6 (3–12)	40.1	8.3
Tongue	6 (3–12)	47.4	21.8
Gum	7 (4–19)	56.1	32.6
Floor of mouth	7 (4–14)	51.0	25.4
Palate	6 (3–14)	47.2	25.6
Other and unspecific parts of mouth	8 (4–12)	57.7	22.5
van Walraven weighted Elixhauser Score			
Low	5 (3–8)	34.3	9.9
Moderate	8 (4–15)	58.2	30.1
High	8 (4–18)	60.6	34.6
Procedures			
Surgical procedures in the oral and facial region	9 (5–17)	68.6	36.4
Operations on the lymphatic system	12 (7–21)	80.5	44.1
Chemotherapy	5 (4–8)	30.5	16.5
Radiotherapy	5 (4–13)	42.2	23.9
Transfusion of blood cells	20 (11–28)	87.3	68.6
Complex intensive care	18 (12–27)	95.9	69.7

## Data Availability

The data used in this study are subject to strict privacy and confidentiality requirements and therefore cannot be made publicly available. Legal restrictions prohibit the sharing of the raw data with third parties.

## Supplementary Materials

The following supporting information can be downloaded at https://www.mdpi.com/article/10.3390/reports9030296/s1, Supplementary Figure S1: Median hospital length of stay (days) across participating hospitals contributing inpatient oral cavity cancer hospitalizations; Supplementary Table S1: Association of demographic and clinical variables with hospital length of stay in patients with oral cavity cancers (univariable Poisson regression models); Supplementary Table S2: Association of demographic and clinical variables with prolonged hospitalization (≥7 and ≥14 days) in patients with oral cavity cancers (univariable logistic regression models); Supplementary Table S3: Age- and sex-adjusted associations between individual Elixhauser comorbidity components and hospital length of stay in inpatient hospitalizations for oral cavity cancer (Poisson regression models with hospital random intercept); Supplementary Table S4: Lip-cancer-excluded sensitivity analysis: comparison of main analysis (full cohort, C00–C06, n = 3957) and lip-cancer-excluded analysis (excluding lip cancer C00, n = 3800) across all three multivariable regression models; Supplementary Table S5: Sensitivity analysis: combined surgical category variable (oral and facial surgery only, lymphatic system operations only, both combined vs. no surgery) across all three multivariable regression models; Supplementary Table S6: Treatment category analysis: associations between treatment modality categories (radiotherapy only, chemotherapy only, combined radiotherapy and chemotherapy, immunotherapy/targeted therapy vs. no systemic therapy) and hospital length of stay; Supplementary Table S7: Exact ICD-10-GM subcode distribution of oral cavity cancer diagnoses in the study cohort (n = 3957 hospitalizations); Supplementary Table S8: Sensitivity analysis restricted to unambiguous oral cavity subsites (excluding base of tongue C01, lingual tonsil C02.4, soft palate C05.1, and uvula C05.2; n = 2961). All models include hospital as a random intercept; Supplementary Table S9: Negative binomial mixed model for hospital length of stay compared with the main Poisson mixed model (n = 3957; hospital random intercept); Supplementary Table S10: Robust (sandwich-variance) Poisson model for hospital length of stay, with standard errors clustered by hospital (n = 3957; 34 clusters).

## References

[1] Sun H., Yu M., An Z., Liang F., Sun B., Liu Y., Zhang S. (2025). Global burden of head and neck cancer: Epidemiological transitions, inequities, and projections to 2050. Front. Oncol..

[2] Ghantous Y., Abu Elnaaj I. (2017). Global incidence and risk factors of oral cancer. Harefuah.

[3] Tan Y., Wang Z., Xu M., Li B., Huang Z., Qin S., Nice E.C., Tang J., Huang C. (2023). Oral squamous cell carcinomas: State of the field and emerging directions. Int. J. Oral Sci..

[4] Robert Koch-Institut, Gesellschaft der epidemiologischen Krebsregister in Deutschland e. V. (2025). Krebs in Deutschland für 2021–2023.

[5] Lindeborg M.M., Sethi R.K.V., Puram S.V., Parikh A., Yarlagadda B., Varvares M., Emerick K., Lin D., Durand M.L., Deschler D.G. (2020). Predicting length of stay in head and neck patients who undergo free flap reconstruction. Laryngoscope Investig. Otolaryngol..

[6] McDevitt J., Cancela M.C., Kelly M., Comber H., Sharp L. (2016). Tracheostomy and infection prolong length of stay in hospital after surgery for head and neck cancer: A population based study. Oral Surg. Oral Med. Oral Pathol. Oral Radiol..

[7] Aponte-Ortiz J.A., Greenberg-Worisek A.J., Marinelli J.P., May M., Spears G.M., Labott J.R., Mecham J.C., Moore E.J., Visscher S.L., Borah B.J. (2021). Cost and clinical outcomes of postoperative intensive care unit versus general floor management in head and neck free flap reconstructive surgery patients. Am. J. Otolaryngol..

[8] Denholm K.A., Steel B.J., Wilson A., Nugent M., Burns A. (2022). Factors determining postoperative length of stay and time to resumption of feeding following free flap reconstruction for oral cancer. Br. J. Oral Maxillofac. Surg..

[9] Gopalkrishnan S., Singhavi H.R., Nair S., Singh A.G., Shetty R., Kannan S., Chaturvedi P. (2024). Predicting length of hospital stay in oral cavity cancer surgery: A nomogram-based approach. J. Head Neck Physicians Surg..

[10] Bhattacharyya N., Fried M.P. (2001). Benchmarks for mortality, morbidity, and length of stay for head and neck surgical procedures. Arch. Otolaryngol. Head Neck Surg..

[11] Nieman C.L., Pitman K.T., Tufaro A.P., Eisele D.W., Frick K.D., Gourin C.G. (2018). The effect of frailty on short-term outcomes after head and neck cancer surgery. Laryngoscope.

[12] van Walraven C., Austin P.C., Jennings A., Quan H., Forster A.J. (2009). A modification of the Elixhauser comorbidity measures into a point system for hospital death using administrative data. Med. Care.

[13] Genther D.J., Gourin C.G. (2015). Effect of comorbidity on short-term outcomes and cost of care after head and neck cancer surgery in the elderly. Head Neck.

[14] Raab G., Restifo D., McBride S.M., Wong R.J., Lee N.Y., Shahrokni A., Zakeri K. (2022). Outcomes following head and neck cancer surgery among older adults as determined by an electronic geriatric assessment. J. Geriatr. Oncol..

[15] Kaur N., Konrad M., Hajek A., Smith L., Kostev K. (2024). Hospital length of stay and associated factors in adult patients with depression in Germany: A cross-sectional study. J. Clin. Med..

[16] Sarabhai T., Jurgens L., Kostev K. (2025). A nationwide analysis of diabetes mellitus and intracranial injuries: No impact on mortality but prolonged hospital stays in Germany. Medicina.

[17] Messerle R., Schreyogg J. (2024). Country-level effects of diagnosis-related groups: Evidence from Germany’s comprehensive reform of hospital payments. Eur. J. Health Econ..

[18] Shaw J.F., Budiansky D., Sharif F., McIsaac D.I. (2022). The association of frailty with outcomes after cancer surgery: A systematic review and meta-analysis. Ann. Surg. Oncol..

[19] Lin H.S., Watts J.N., Peel N.M., Hubbard R.E. (2016). Frailty and post-operative outcomes in older surgical patients: A systematic review. BMC Geriatr..

[20] Handforth C., Clegg A., Young C., Simpkins S., Seymour M.T., Selby P.J., Young J. (2015). The prevalence and outcomes of frailty in older cancer patients: A systematic review. Ann. Oncol..

[21] Schwartz C., Ueberschaer M.F., Rautalin I., Grauvogel J., Bissolo M., Masalha W., Steiert C., Schnell O., Beck J., Ebel F. (2024). Frailty indices predict mortality, complications and functional improvements in supratentorial meningioma patients over 80 years of age. J. Neurooncol..

[22] Meerkerk C.D.A., Chargi N., de Jong P.A., van den Bos F., de Bree R. (2022). Low skeletal muscle mass predicts frailty in elderly head and neck cancer patients. Eur. Arch. Otorhinolaryngol..

[23] Dewansingh P., Bras L., Ter Beek L., Krijnen W.P., Roodenburg J.L.N., van der Schans C.P., Halmos G.B., Jager-Wittenaar H. (2023). Malnutrition risk and frailty in head and neck cancer patients: Coexistent but distinct conditions. Eur. Arch. Otorhinolaryngol..

[24] Wieland M.W.M., Pilz W., Winkens B., Hoeben A., Willemsen A.C.H., Kremer B., Baijens L.W.J. (2023). Multi-domain screening: Identification of patient’s risk profile prior to head-and-neck cancer treatment. Cancers.

[25] de Bree R., Meerkerk C.D.A., Halmos G.B., Mäkitie A.A., Homma A., Rodrigo J.P., López F., Takes R.P., Vermorken J.B., Ferlito A. (2022). Measurement of sarcopenia in head and neck cancer patients and its association with frailty. Front. Oncol..

[26] Mendes M.L., Mahl C., Carvalho A.C., Santos V.S., Tanajura D.M., Martins-Filho P.R. (2021). Frailty and risk of complications in head and neck oncologic surgery: Systematic review and dose-response meta-analysis. Med. Oral Patol. Oral Cir. Bucal.

[27] Schmitz A., Reichardt C., Gierth J., Richter M., Mrosk F., Alfertshofer M., Knoedler L., Doll C., Hausmann F., Lee M. (2025). Retrospective analysis of multimorbidity, polypharmacy, and drug interactions on postoperative outcomes in oral squamous cell carcinoma patients. Clin. Oral Investig..

[28] Rosen C.B., Roberts S.E., Wirtalla C.J., Ramadan O.I., Keele L.J., Kaufman E.J., Halpern S.D., Kelz R.R. (2022). Analyzing impact of multimorbidity on long-term outcomes after emergency general surgery: A retrospective observational cohort study. J. Am. Coll. Surg..

[29] Han H.R., Hermann G.M., Ma S.J., Iovoli A.J., Wooten K.E., Arshad H., Gupta V., McSpadden R.P., Kuriakose M.A., Markiewicz M.R. (2020). Matched pair analysis to evaluate the impact of hospitalization during radiation therapy as an early marker of survival in head and neck cancer patients. Oral Oncol..

[30] Foote R.L., Harmsen W.S., Amundson A.C., Carr A.B., Gamez M.E., Garces Y.I., Lester S.C., Ma D.J., McGee L.A., Moore E.J. (2024). Mean oral cavity organ-at-risk dose predicts opioid use and hospitalization during radiotherapy for patients with head and neck tumors. Cancers.

[31] Ferris R.L., Blumenschein G., Fayette J., Guigay J., Colevas A.D., Licitra L., Harrington K., Kasper S., Vokes E.E., Even C. (2016). Nivolumab for recurrent squamous-cell carcinoma of the head and neck. N. Engl. J. Med..

[32] Bonner J.A., Harari P.M., Giralt J., Azarnia N., Shin D.M., Cohen R.B., Jones C.U., Sur R., Raben D., Jassem J. (2006). Radiotherapy plus cetuximab for squamous-cell carcinoma of the head and neck. N. Engl. J. Med..

