# Supplementary material for: Hospital Length of Stay and Associated Factors in Patients with Oral Cavity Cancer in Germany: A Retrospective Multicenter Analysis of Inpatient Administrative Data
Source: Reports (MDPI). 2026 Sep 2;9(3):296. doi: 10.3390/reports9030296 (PMC13611555; doi:10.3390/reports9030296)
Supplement: Supplementary file 1 [file reports-09-00296-s001.zip › reports-4518769-supplementary.pdf]

Table S1. Association of demographic and clinical variables with hospital length of stay in patients with oral cavity cancers (univariable Poisson regression models)

Variable	Rate ratio (95% CI)	P value
Age		
≤60 years	Reference	
61-70 years	1.00 (0.98-1.03)	0.730
71-80 years	1.13 (1.10-1.16)	<0.001
>80 years	1.14 (1.11-1.18)	<0.001
Sex		
Female	Reference	
Male	1.01 (0.99-1.03)	0.419
Primary cancer diagnosis		
Lip	0.77 (0.73-0.82)	<0.001
Tongue	Reference	
Gum	1.36 (1.32-1.41)	<0.001
Floor of mouth	1.18 (1.15-1.21)	<0.001
Palate	1.15 (1.10-1.19)	<0.001
Other and unspecific parts of mouth	1.10 (1.05-1.14)	<0.001
van Walraven weighted Elixhauser Score		
Low	Reference	
Moderate	1.75 (1.71-1.80)	<0.001
High	2.04 (1.99-2.09)	<0.001
Procedures		
Surgical procedures in the oral and facial region	1.20 (1.17-1.24)	<0.001
Operations on the lymphatic system	1.30 (1.26-1.34)	<0.001
Chemotherapy	0.86 (0.83-0.89)	<0.001
Radiotherapy	1.88 (1.83-1.94)	<0.001
Transfusion of blood cells	2.01 (1.97-2.07)	<0.001
Complex intensive care	1.79 (1.74-1.84)	<0.001

Table S2. Association of demographic and clinical variables with prolonged hospitalization (≥7 and ≥14 days) in patients with oral cavity cancers (univariable logistic regression models)

	≥7 days		≥14 days	
Variable	Odds ratio (95% CI)	P value	Odds ratio (95% CI)	P value
Age				
Age ≤60 years	Reference		Reference	
Age 61-70 years	0.97 (0.84-1.13)	0.734	1.02 (0.86-1.23)	0.755
Age 71-80 years	1.17 (0.98-1.40)	0.082	1.19 (0.97-1.47)	0.094
Age >80 years	1.44 (1.16-1.80)	0.001	1.30 (1.02-1.67)	0.037
Sex, n (%)				
Female	Reference		Reference	
Male	0.97 (0.85-1.11)	0.641	0.96 (0.83-1.13)	0.641
Primary cancer diagnosis				
Lip	0.74 (0.53-1.04)	0.079	0.32 (0.18-0.58)	<0.001
Tongue	Reference		Reference	
Gum	1.42 (1.15-1.75)	0.001	1.74 (1.38-2.19)	<0.001
Floor of mouth	1.15 (0.98-1.36)	0.086	1.22 (1.01-1.48)	0.040
Palate	0.99 (0.78-1.26)	0.933	1.24 (0.64-1.63)	0.127
Other and unspecific parts of mouth	1.51 (1.16-1.96)	0.002	1.04 (0.76-1.43)	0.787
van Walraven weighted Elixhauser Score				
Low	Reference		Reference	
Moderate	2.67 (2.26-3.13)	<0.001	3.91 (3.16-4.83)	<0.001
High	2.95 (2.54-3.43)	<0.001	4.80 (3.94-5.86)	<0.001
Procedures				
Surgical procedures in the oral and facial region	1.54 (1.29-1.85)	<0.001	1.51 (1.20-1.89)	<0.001
Operations on the lymphatic system	3.63 (2.95-4.47)	<0.001	2.23 (1.76-2.83)	<0.001
Chemotherapy	0.42 (0.33-0.55)	<0.001	0.59 (0.43-0.80)	<0.001
Radiotherapy	2.27 (1.82-2.83)	<0.001	3.28 (2.51-4.29)	<0.001
Transfusion of blood cells	5.92 (4.34-8.07)	<0.001	7.12 (5.56-9.10)	<0.001
Complex intensive care	12.17 (7.78-19.06)	<0.001	5.37 (4.21-6.85)	<0.001

Figure S1. Median hospital length of stay (days) across participating hospitals contributing inpatient oral cavity cancer hospitalizations.

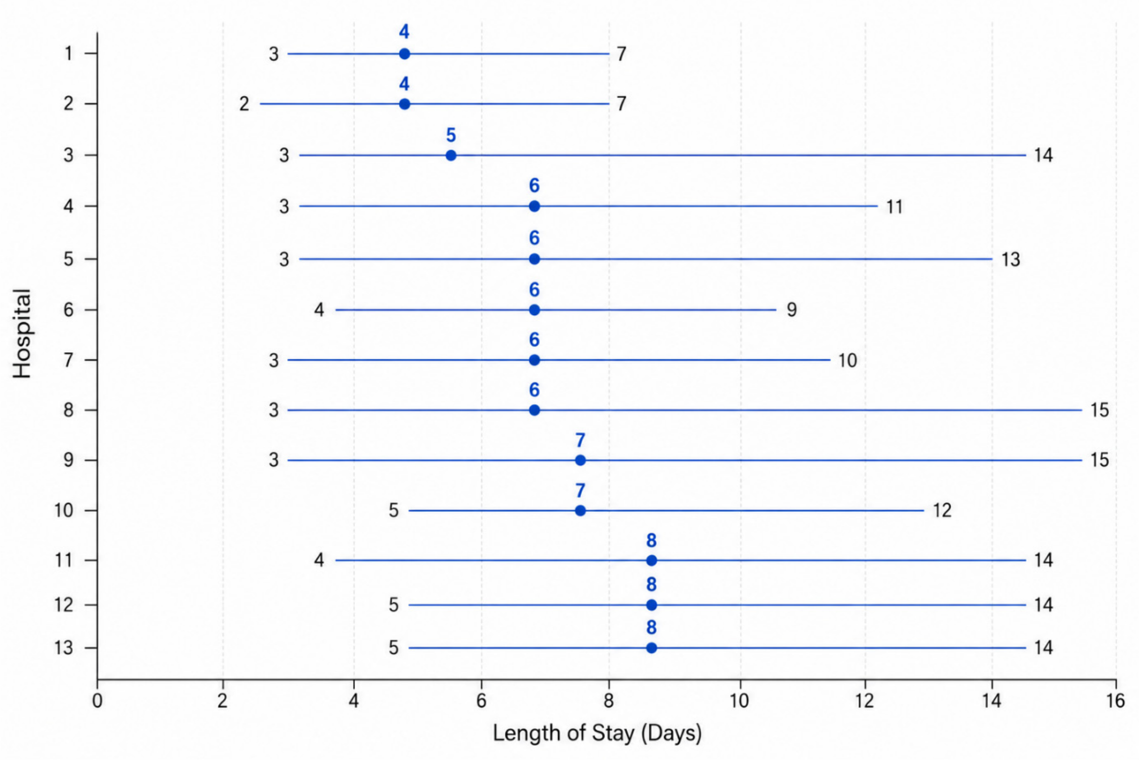

Table S3. Age- and sex-adjusted associations between individual Elixhauser comorbidity components and hospital length of stay in inpatient hospitalizations for oral cavity cancer (Poisson regression models with hospital random intercept).

Comorbidity	van Walraven weight	RR	95% CI	p-value
Coagulopathy	3	2.91	2.78–3.03	<0.001
Fluid and electrolyte disorders	5	2.18	2.13–2.22	<0.001
Congestive heart failure	7	1.97	1.90–2.04	<0.001
Valvular disease	4	1.95	1.84–2.07	<0.001
Pulmonary circulation disorders	6	1.88	1.76–2.00	<0.001
Weight loss	6	1.86	1.80–1.92	<0.001
Depression	−3	1.46	1.40–1.52	<0.001
Cardiac arrhythmias	5	1.46	1.41–1.50	<0.001
Liver disease	11	1.45	1.38–1.53	<0.001
Metastatic cancer	14	1.41	1.38–1.44	<0.001
Renal failure	5	1.35	1.30–1.40	<0.001
Other neurological disorders	6	1.31	1.21–1.43	<0.001
Complicated diabetes	2	1.28	1.20–1.38	<0.001
Solid tumor without metastasis	4	1.19	1.15–1.23	<0.001
Peripheral vascular disease	2	1.16	1.12–1.21	<0.001
Chronic pulmonary disease	3	1.09	1.05–1.13	<0.001
Paralysis	5	1.04	0.97–1.12	0.257
Lymphoma (not present in cohort)	*9*	—	—	—

RR = Rate Ratio; CI = 95% Confidence Interval. Each comorbidity was modeled in a separate Poisson regression model adjusted for age category and sex, with hospital as a random intercept. Results are sorted by rate ratio in descending order. van Walraven weights reflect the contribution of each comorbidity to the overall Elixhauser comorbidity score. Lymphoma was not present in the cohort and could not be estimated.

Table S4. Lip-cancer-excluded sensitivity analysis: comparison of main analysis (full cohort, C00–C06, n=3,957) and lip-cancer-excluded analysis (excluding lip cancer C00, n=3,800) across all three multivariable regression models.

Variable	Main analysis	Main (95% CI)	p	OSCC (95% CI)	p	n (full)
**A. Poisson regression — Rate Ratios (LOS continuous)**						
Comorbidity moderate vs. low	3,957	1.38 (1.34–1.42)	<0.001	1.42 (1.38–1.46)	<0.001	3,800
Comorbidity high vs. low	3,957	1.58 (1.54–1.63)	<0.001	1.74 (1.69–1.79)	<0.001	3,800
Transfusion	3,957	1.80 (1.76–1.85)	<0.001	1.76 (1.71–1.81)	<0.001	3,800
Intensive care	3,957	1.70 (1.65–1.75)	<0.001	1.65 (1.61–1.70)	<0.001	3,800
Radiotherapy	3,957	1.77 (1.71–1.82)	<0.001	1.70 (1.65–1.76)	<0.001	3,800
Chemotherapy	3,957	0.85 (0.82–0.88)	<0.001	0.85 (0.82–0.88)	<0.001	3,800
**B. Logistic regression — Odds Ratios (LOS ≥7 days)**						
Comorbidity moderate vs. low	3,957	2.12 (1.75–2.57)	<0.001	2.18 (1.83–2.60)	<0.001	3,800
Comorbidity high vs. low	3,957	2.42 (2.00–2.93)	<0.001	4.13 (3.26–5.24)	<0.001	3,800
Transfusion	3,957	4.80 (3.49–6.62)	<0.001	4.59 (3.32–6.33)	<0.001	3,800
Intensive care	3,957	10.63 (6.74–16.78)	<0.001	9.80 (6.20–15.51)	<0.001	3,800
**C. Logistic regression — Odds Ratios (LOS ≥14 days)**						
Comorbidity moderate vs. low	3,957	2.71 (2.12–3.45)	<0.001	2.53 (2.03–3.16)	<0.001	3,800
Comorbidity high vs. low	3,957	3.87 (3.05–4.91)	<0.001	5.84 (4.48–7.62)	<0.001	3,800
Transfusion	3,957	5.70 (4.40–7.38)	<0.001	5.49 (4.22–7.14)	<0.001	3,800
Intensive care	3,957	5.12 (3.93–6.66)	<0.001	4.79 (3.66–6.26)	<0.001	3,800

Selected key variables shown; full table available in TableS4_OSCC_Sensitivity.docx. RR = Rate Ratio; OR = Odds Ratio; CI = 95% Confidence Interval. All models include hospital as random intercept. Reference categories: comorbidity low; no respective procedure.

Table S5. Sensitivity analysis: combined surgical category variable (oral and facial surgery only, lymphatic system operations only, both combined vs. no surgery) across all three multivariable regression models.

Variable	n	Poisson RR (95% CI)	p	LOS ≥7d OR (95% CI)	p	LOS ≥14d OR (95% CI)
**No surgery (reference)**	2,437	*Reference*	—	*Reference*	—	*Reference*
Oral and facial surgery only	526	1.30 (1.26–1.35)	<0.001	1.20 (0.95–1.51)	0.130	1.56 (1.14–2.15)
Lymphatic system operations only	247	1.24 (1.19–1.30)	<0.001	1.24 (0.90–1.70)	0.191	1.34 (0.90–1.98)
Both procedures combined	747	1.60 (1.55–1.64)	<0.001	9.59 (7.23–12.72)	<0.001	4.19 (3.21–5.48)

All models include hospital as random intercept. Reference category for surgical variable: no surgery (n=2,437). Poisson model estimated with method=Laplace. RR = Rate Ratio; OR = Odds Ratio; CI = 95% Confidence Interval. p-values for the ≥14 days logistic model: oral and facial surgery only p=0.006; lymphatic operations only p=0.146; both combined p<0.001.

Table S6. Treatment category analysis: associations between treatment modality categories (radiotherapy only, chemotherapy only, combined radiotherapy and chemotherapy, immunotherapy/targeted therapy vs. no systemic therapy) and hospital length of stay.

Treatment category	n	Median LOS	Poisson RR (95% CI)	p	LOS ≥7d OR (95% CI)	p	LOS ≥14d OR (95% CI)	p
No systemic therapy (ref.)	2,956	7	*Reference*	—	*Reference*	—	*Reference*	—
Radiotherapy only	344	8	1.75 (1.69–1.81)	<0.001	2.08 (1.59–2.72)	<0.001	2.52 (1.84–3.44)	<0.001
Chemotherapy only	72	5	1.08 (1.00–1.17)	0.050	0.56 (0.32–0.97)	0.038	0.69 (0.34–1.39)	0.301
Radiotherapy + Chemotherapy	503	5	1.48 (1.43–1.54)	<0.001	0.67 (0.52–0.86)	0.002	1.32 (0.96–1.82)	0.084
Immunotherapy / targeted therapy	82	7.5	1.89 (1.80–1.99)	<0.001	0.68 (0.39–1.20)	0.185	1.68 (0.87–3.24)	0.119

RR = Rate Ratio; OR = Odds Ratio; CI = 95% Confidence Interval. Immunotherapy/targeted therapy (OPS 6-002) assigned as priority category. All models include hospital as random intercept. Reference category: no systemic therapy (n=2,956).

Immunotherapy/targeted therapy (OPS 6-002; n=82, 2.1%) was assigned as priority category. All models include hospital as random intercept. RR = Rate Ratio; OR = Odds Ratio; CI = 95% Confidence Interval.

Table S7. Exact ICD-10-GM subcode distribution of oral cavity cancer diagnoses in the study cohort (n=3,957 hospitalizations).

ICD-10-GM code	Description	n	%
**C00.0**	Malignant neoplasm of external upper lip	13	0.37
**C00.1**	Malignant neoplasm of external lower lip	101	2.86
**C00.3**	Malignant neoplasm of upper lip, inner aspect	3	0.08
**C00.4**	Malignant neoplasm of lower lip, inner aspect	7	0.20
**C00.5**	Malignant neoplasm of lip, unspecified, inner aspect	1	0.03
**C00.6**	Malignant neoplasm of commissure of lip	2	0.06
**C00.8**	Overlapping lesion of lip	6	0.17
**C00.9**	Malignant neoplasm of lip, unspecified	5	0.14
**C01 ***	Malignant neoplasm of base of tongue (oropharynx)	739	20.92
**C02.0**	Dorsal surface of tongue	19	0.54
**C02.1**	Border of tongue	712	20.15
**C02.2**	Ventral surface of tongue	44	1.25
**C02.3**	Anterior two-thirds of tongue, part unspecified	23	0.65
**C02.4 ***	Lingual tonsil (oropharynx)	11	0.31
**C02.8**	Overlapping lesion of tongue	164	4.64
**C02.9**	Tongue, unspecified	47	1.33
**C03.0**	Upper gum	99	2.80
**C03.1**	Lower gum	260	7.36
**C03.9**	Gum, unspecified	2	0.06
**C04.0**	Anterior floor of mouth	195	5.52
**C04.1**	Lateral floor of mouth	118	3.34
**C04.8**	Overlapping lesion of floor of mouth	284	8.04
**C04.9**	Floor of mouth, unspecified	153	4.33
**C05.0**	Hard palate	43	1.22
**C05.1 ***	Soft palate (oropharynx)	104	2.94
**C05.2 ***	Uvula (oropharynx)	29	0.82
**C05.8**	Overlapping lesion of palate	81	2.29
**C05.9**	Palate, unspecified	28	0.79
**C06.0**	Cheek mucosa	132	3.74
**C06.1**	Vestibule of mouth	11	0.31
**C06.2**	Retromolar area	27	0.76
**C06.8**	Overlapping lesion of other/unspecified mouth	26	0.74
**C06.9**	Mouth, unspecified	44	1.25

*Percentages are calculated over 3,533 coded oral cavity cancer diagnoses; a single hospitalization may carry more than one code. * Subsites marked with an asterisk anatomically belong to the oropharynx and were excluded in the sensitivity analysis restricted to unambiguous oral cavity subsites (Table S8).*

Table S8. Sensitivity analysis restricted to unambiguous oral cavity subsites (excluding base of tongue C01, lingual tonsil C02.4, soft palate C05.1, and uvula C05.2; n=2,961). All models include hospital as a random intercept.

Variable	Poisson RR (95% CI)	LOS >=7 d OR (95% CI)	LOS >=14 d OR (95% CI)

**Moderate vs low comorbidity**	1.45 (1.41-1.49)	2.14 (1.75-2.61)	2.82 (2.19-3.63)
**High vs low comorbidity**	1.73 (1.68-1.79)	4.30 (3.26-5.67)	6.57 (4.82-8.95)
**Age 61-70 vs <=60 y**	0.98 (0.95-1.01)	1.06 (0.86-1.31)	1.04 (0.80-1.34)
**Age 71-80 vs <=60 y**	0.98 (0.95-1.01)	1.13 (0.86-1.47)	0.90 (0.66-1.24)
**Age >80 vs <=60 y**	1.16 (1.11-1.20)	1.57 (1.16-2.13)	1.58 (1.10-2.27)
**Male vs female**	1.11 (1.08-1.14)	1.05 (0.87-1.27)	1.16 (0.92-1.45)
**Transfusion of blood cells**	1.75 (1.70-1.80)	4.27 (3.01-6.05)	5.76 (4.31-7.70)
**Complex intensive care**	1.63 (1.58-1.68)	14.41 (8.06-25.75)	5.31 (3.94-7.15)
**Oral/facial surgery**	1.34 (1.30-1.39)	1.85 (1.47-2.33)	2.04 (1.53-2.72)
**Lymphatic system operations**	1.20 (1.16-1.24)	3.54 (2.74-4.59)	1.84 (1.36-2.48)
**Chemotherapy**	0.91 (0.87-0.95)	0.40 (0.30-0.55)	0.66 (0.46-0.96)
**Radiotherapy**	1.67 (1.62-1.73)	2.08 (1.57-2.74)	2.36 (1.70-3.27)
**Lip (C00) vs tongue**	0.87 (0.82-0.93)	0.74 (0.49-1.12)	0.35 (0.18-0.70)
**Gum (C03) vs tongue**	1.10 (1.06-1.14)	1.10 (0.82-1.48)	1.29 (0.92-1.80)
**Floor of mouth (C04) vs tongue**	1.06 (1.03-1.09)	0.87 (0.70-1.10)	1.01 (0.77-1.32)
**Palate (C05) vs tongue**	1.16 (1.10-1.22)	0.91 (0.62-1.33)	1.33 (0.84-2.09)
**Other/unspecified (C06) vs tongue**	0.99 (0.95-1.03)	1.23 (0.88-1.72)	0.74 (0.49-1.12)

RR = rate ratio; OR = odds ratio; CI = confidence interval. Reference categories: low comorbidity burden; age <=60 years; female sex; tongue (oral tongue); no respective procedure. Associations are consistent in direction, magnitude, and significance with the main analysis.

Table S9. Negative binomial mixed model for hospital length of stay compared with the main Poisson mixed model (n=3,957; hospital random intercept).

Variable	Poisson RR (95% CI)	Negative binomial RR (95% CI)
**Moderate vs low comorbidity**	1.38 (1.34-1.42)	1.40 (1.33-1.48)
**High vs low comorbidity**	1.58 (1.54-1.63)	1.74 (1.63-1.85)
**Age 61-70 vs <=60 y**	1.00 (0.98-1.03)	1.00 (0.95-1.06)
**Age 71-80 vs <=60 y**	1.03 (1.00-1.06)	1.04 (0.98-1.11)
**Age >80 vs <=60 y**	1.17 (1.13-1.21)	1.16 (1.07-1.26)
**Male vs female**	1.09 (1.07-1.12)	1.07 (1.02-1.12)
**Transfusion of blood cells**	1.80 (1.76-1.85)	1.84 (1.72-1.97)
**Complex intensive care**	1.70 (1.65-1.75)	1.64 (1.53-1.76)
**Oral/facial surgery**	1.29 (1.25-1.32)	1.24 (1.17-1.32)
**Lymphatic system operations**	1.23 (1.20-1.27)	1.32 (1.24-1.41)
**Chemotherapy**	0.85 (0.82-0.88)	0.81 (0.75-0.88)
**Radiotherapy**	1.77 (1.71-1.82)	1.66 (1.55-1.78)
**Lip (C00) vs tongue**	0.88 (0.83-0.94)	0.82 (0.73-0.93)
**Gum (C03) vs tongue**	1.10 (1.06-1.13)	1.11 (1.03-1.20)
**Floor of mouth (C04) vs tongue**	1.06 (1.03-1.09)	1.03 (0.98-1.10)
**Palate (C05) vs tongue**	1.15 (1.10-1.19)	1.09 (1.00-1.18)
**Other/unspecified (C06) vs tongue**	0.99 (0.95-1.03)	1.02 (0.93-1.12)

RR = rate ratio; CI = confidence interval. Marginal Poisson model dispersion: Deviance/df = 5.18, Pearson chi-square/df = 6.69. Negative binomial model: conditional Pearson chi-square/df = 1.33; scale (dispersion) parameter 0.36; hospital-intercept variance 0.046 (p=0.010). Estimates are consistent in direction and statistical significance with the Poisson model, with slightly wider confidence intervals.

Table S10. Robust (sandwich-variance) Poisson model for hospital length of stay, with standard errors clustered by hospital (n=3,957; 34 clusters).

Variable	Robust Poisson RR (95% CI)	p-value
**Moderate vs low comorbidity**	1.41 (1.21–1.64)	<0.001
**High vs low comorbidity**	1.67 (1.50–1.86)	<0.001
**Age 61-70 vs ≤60 y**	1.01 (0.89–1.14)	0.911
**Age 71-80 vs ≤60 y**	1.03 (0.92–1.14)	0.662
**Age >80 vs ≤60 y**	1.13 (1.05–1.22)	0.001
**Male vs female**	1.06 (1.00–1.13)	0.035
**Transfusion of blood cells**	1.82 (1.44–2.29)	<0.001
**Complex intensive care**	1.64 (1.51–1.77)	<0.001
**Oral/facial surgery**	1.30 (1.12–1.50)	<0.001
**Lymphatic system operations**	1.21 (1.02–1.45)	0.032
**Chemotherapy**	0.86 (0.75–1.00)	0.045
**Radiotherapy**	1.76 (1.53–2.02)	<0.001
**Lip (C00) vs tongue**	0.90 (0.78–1.05)	0.169
**Gum (C03) vs tongue**	1.09 (1.00–1.19)	0.050
**Floor of mouth (C04) vs tongue**	1.05 (0.98–1.12)	0.178
**Palate (C05) vs tongue**	1.10 (0.88–1.37)	0.423
**Other/unspecified (C06) vs tongue**	0.98 (0.89–1.08)	0.712

RR = rate ratio; CI = confidence interval. Robust (empirical sandwich-variance) Poisson model estimated by generalized estimating equations with a working-independence correlation structure and hospital as the clustering unit (34 clusters). Point estimates correspond to the marginal Poisson model; the confidence intervals are robust to overdispersion and to within-hospital clustering. All principal associations remained statistically significant. Reference categories: low comorbidity burden; age ≤60 years; female sex; tongue; no respective procedure.
